# The effect of synbiotic supplementation on plasma levels of advanced glycation end products and cardiovascular risk factors in hemodialysis patients: A double‐blind clinical trial

**DOI:** 10.1002/fsn3.4338

**Published:** 2024-07-12

**Authors:** Yasaman Azamian, Hadi Abdollahzad, Shahab Rezaeian, Mohammad Hossein Rouhani, Mohammad Hossein Fatehi

**Affiliations:** ^1^ Student Research Committee, School of Nutritional Sciences and Food Technology Kermanshah University of Medical Sciences Kermanshah Iran; ^2^ Department of Nutrition, School of Medicine Urmia University of Medical Sciences Urmia Iran; ^3^ Infectious Diseases Research Center, Health Institute Kermanshah University of Medical Sciences Kermanshah Iran; ^4^ Nutrition and Food Security Research Center, Department of Community Nutrition, School of Nutrition and Food Science Isfahan University of Medical Sciences Isfahan Iran; ^5^ Department of Internal Medicine, Farabi Hospital Isfahan University of Medical Sciences Isfahan Iran

**Keywords:** advanced glycation end products, dysbiosis, fibrinogen, hemodialysis, synbiotic

## Abstract

There is increasing evidence supporting the relationship between imbalance of gut microbiota and development of chronic kidney and cardiovascular diseases. This study aimed to investigate the effect of synbiotic supplementation on plasma levels of advanced glycation end products (AGEs) and cardiovascular risk factors in hemodialysis (HD) patients. In this randomized, double‐blind, placebo‐controlled clinical trial, 36 HD patients were randomly allocated into two groups to receive two synbiotic supplements (*n* = 19) or placebo (*n* = 17) daily for 12 weeks. Levels of AGEs, fibrinogen, hemoglobin A1c (HbA1c), and other measures were assessed at the beginning and end of the study. The data were analyzed using independent t‐tests, paired t‐tests, and analysis of covariance (ANCOVA). At the end of the study, the plasma levels of AGEs increased significantly in both the synbiotic (*p* < .001) and control (*p* = .001) groups, but the difference between the groups was not significant (*p* = .272). Plasma levels of fibrinogen decreased specifically within the synbiotic group (*p* = .007), and a statistically significant disparity between the groups persisted at the study's conclusion (*p* = .016). The mean levels of blood urea nitrogen (BUN) decreased (*p* < .05) in both groups, but there was no difference between the two groups at the end of the study (*p* = .116). No significant differences were observed in other measured biomarkers. Synbiotic supplementation improved plasma fibrinogen and BUN levels in HD patients, but did not significantly improve AGEs and HbA1c. Further investigations are needed to investigate the effect of probiotics on AGEs in HD patients at different stages of kidney disease.

## Graphical abstract

Synbiotic supplementation for 12 weeks significantly decreased the plasma levels of fibrinogen; however, it had no effect on advanced glycogen end products (AGEs), hemoglobin A1c (HbA1c), and blood urea nitrogen (BUN) in hemodialysis (HD) patients.

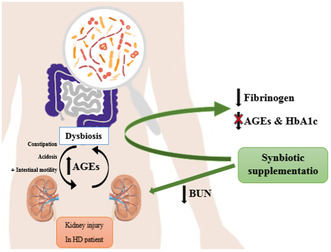

## 1 INTRODUCTION

Chronic kidney disease (CKD) arises from various disease pathways leading to irreversible changes in kidney function and structure over an extended duration. The glomerular filtration rate (GFR) is regarded as the most dependable measure of overall kidney function (Levey et al., 2015). The last stage of CKD requires alternative treatment, which is usually Hemodialysis (HD) treatment. In End‐stage renal disease patients (ESRD), the GFR is lower than 50% normal (Sesso et al., 2014). More than 50% of HD patients have been reported to experience cardiovascular disease (CVD), with the relative risk of mortality from CVD events in this patient group reported to be 20 times higher than that in the general population (Cozzolino et al., 2017). The HD patients exhibit disease‐specific risk factors for CVD, including anemia, hyperhomocysteinemia, undernutrition, and chronic inflammation (Gansevoort et al., 2013; Herzog et al., 2011). AGEs can contribute to the advancement of atherosclerosis by engaging with receptors, resulting in the generation of reactive oxygen species (ROS) and the secretion of inflammatory cytokines across various cell types (Bierhaus et al., 1998; Raj et al., 2000). Since the kidney plays a crucial role in the elimination of AGEs, the gradual decline in kidney function results in the buildup of AGEs in individuals with chronic kidney failure (Gugliucci & Bendayan, 1996; Miyata et al., 1998, 1999).

Advanced glycation end products (AGEs) in the human body primarily result from glycosylation (Henning & Glomb, 2016). This nonenzymatic reaction consists of a Schiff base and the attachment of a glucose molecule or other sugars to an amino acid or free protein, which is then converted to a ketoamine called “Amadori product.” This relatively inert compound has the potential to undergo subsequent chemical transformations, including oxidation and dehydration, leading to the formation of AGEs. These AGEs are present in long‐lived extracellular matrix proteins, short‐lived plasma proteins like serum albumin, and intracellular proteins, as well as being derived from dietary intake and intestinal absorption (Koschinsky et al., 1997; Schepers et al., 2010). AGEs exist as free compounds in the bloodstream and urine (Alamir et al., 2013). Elevated levels of AGEs represent a risk factor for CVD and serve as a prognostic indicator for mortality in HD (Zimmermann et al., 1999). Research findings suggest that the interplay between AGEs and receptors for advanced glycation end products (RAGE) results in oxidative stress, triggering inflammatory, thrombogenic, and fibrotic responses. Hence, AGEs may contribute to age‐related conditions, such as CVD and atherosclerosis (Schmidt & Stern, 2000; Ward et al., 2013). Moreover, the aggregation of AGEs in the gastrointestinal tract of individuals with CKD is linked to dysbiosis of the gut microbiota and may potentially contribute to the continued advancement of CKD (Yuan et al., 2018). Recent research has demonstrated that dysbiosis is a pivotal factor in hastening the advancement of CKD (Jazani et al., 2019). Some intestinal variations, such as alterations in intestinal mutations favoring proteolytic microorganisms, abnormal movements of the intestine, metabolic acidosis, uremic toxin accumulation, frequent antibiotic administration, and oral iron consumption in the gastrointestinal tract, may increase the production of toxins and be related to renal failure (Ramezani et al., 2016; Ramezani & Raj, 2014; Strid et al., 2003; Vaziri et al., 2013; Wu et al., 2004). It is assumed that an improved intestinal microbiota can reduce circulating AGEs levels and improve metabolic health in HD patients.

Probiotics, prebiotics, and synbiotics aid in the administration of CKD by ameliorating intestinal dysbiosis and reducing uremic toxin concentrations (Joossens et al., 2019; Koppe et al., 2015). Several systematic reviews and meta‐analyses support the gut–kidney axis theory and have shown that probiotic supplementation improves C‐reactive protein (CRP), oxidative stress biomarkers, and lipid profiles in CKD populations (Bakhtiary et al., 2021; Zheng et al., 2021). Therefore, the use of probiotics to inhibit the interactions between dysbiosis and increase the aggregation of AGEs in ESRD is promising. Studies have shown that lactobacilli strains are effective in improving uremia products. However, the protective effect of probiotic bacteria against AGE aggregation in HD patients has not been investigated so far. Due to the high rate of AGEs in ESRD patients, the elevated incidence of cardiovascular disease, and consequently the heightened mortality rate among this patient population, this study aimed to evaluate the effect of synbiotic supplementation on plasma levels of advanced glycation end products and cardiovascular risk factors in HD patients.

## 2 METHOD

### 2.1 Subjects

Patients undergoing hemodialysis were included in the study regardless of age, sex, and race, as long as they met specific criteria: receiving hemodialysis at least twice a week for 6 months, with each session lasting up to 4 hours; having no immunodeficiency; no history of cancers; not lactating or pregnant; free of acute medical conditions, such as cardiovascular, liver diseases, and acute pancreatitis; not using synbiotics, probiotics, prebiotics, or antibiotics in the 4 weeks before the study; no alcohol addiction or consumption; absence of severe gastrointestinal disorders and diseases, HIV (human immunodeficiency virus) disease, or psychological problems; and the ability to drink at least 200 mL of water daily. Additionally, patients with severe edema, those who were candidates for kidney transplantation, transplantation of other organs, or peritoneal dialysis during the study, and patients who experienced adverse effects due to the consumption of supplements were excluded.

Before starting the study, we assessed 50 HD patients to establish the sample size in prior research (Yacoub et al., 2017). A minimum sample size of 15 participants per group was deemed necessary for the study. Nevertheless, to accommodate potential attrition, the sample size was augmented to 21 individuals in each group. Written informed consent forms were completed and signed by the patients. The randomized, double‐blind, placebo‐controlled clinical trial was carried out at Farabi Hospital's dialysis department in Isfahan, from December to February 2022.

### 2.2 Study design

After obtaining informed consent from the participants, all subjects were required to complete a questionnaire regarding their demographic features, smoking habits, health conditions, medical history, and present drug use. The participants were then randomly assigned to two groups: the synbiotic (SYN) group (*n* = 21) and the control (CON) group (*n* = 21) using RAS (remote access service) software, taking into account their age and gender. Both the patients and the researchers were blinded to the process.

For this study, the supplement company encoded the placebo and synbiotic capsules as A and B. The supplement group received two synbiotic capsules daily (GeriLact Brand; Zist Takhmir Co., Tehran, Iran) amount of 10^9^ colony‐forming units (CFU) probiotics containing *Lactobacillus rhamnosus*, *Lactobacillus casei*, *Lactobacillus acidophilus*, *Lactobacillus bulgaricus*, *Lactobacillus fermentum*, *Lactobacillus plantarum*, *Lactobacillus gasseri*, and 21 mg fructooligosaccharides as prebiotic and CON groups received two capsules per day (each capsule containing 350 mg of inulin, maltodextrin, and all of the other ingredients contained in the synbiotic product, except the effective substance) after lunch and dinner for 12 weeks. Subjects' compliance with supplements or placebo was assessed by weekly telephone calls. Patients were requested to disclose their consumption of probiotic‐containing foods, including cheese, kefir, and yogurt, with the consumption of these foods being regarded as a potential confounding factor at the end of the study.

Data on patients' food intake, physical activity, height, weight, and body mass index (BMI) were collected at the beginning and end of the study. Following the requisite training, participants completed a dietary intake assessment spanning 3 days (2 dialysis days and 1 nondialysis day) utilizing a food record questionnaire, with an interview conducted to validate the precision of recorded data. The Persian version of the International Physical Activity Questionnaire (IPAQ) was utilized to evaluate the levels of physical activity. Acceptable validity and reliability were confirmed (Hallal & Victora, 2004). The questionnaire consists of seven questions and considers whether the patient engaged in vigorous, moderate exercise, or walking for at least 10 min over 7 days.

The primary outcome of this study involved the assessment of plasma levels of AGEs, and secondary outcomes were the measurement of plasma fibrinogen, fasting blood sugar (FBS), hemoglobin A1c, creatinine (Cr), blood urea nitrogen (BUN), hemoglobin (Hb), sodium (Na), potassium (K), calcium (Ca), phosphorus (P), cholesterol, and triglycerides (TGs) (beginning and end of the study by using blood samples after a 12‐h fast before dialysis). Blood HbA1c levels were estimated using an enzyme‐corrected method up to 1 h after blood collection, and plasma fibrinogen levels were measured using a kit up to 3 h after blood collection according to the Claus method. Plasma samples were stored at −80°C for AGE level determination. Plasma levels of AGEs were quantified using a human enzyme‐linked immunosorbent assay (ELISA) kit (ZellBio GmbH, Germany).

### 2.3 Statistical analysis

To analyze the data in food record questionnaires, after converting the values to grams, Nutritionist IV Software (Version 4.1, 1997; First DataBank, San Bruno, CA) was used for Iranian foods and SPSS version 26 was used for statistical analysis. The data were analyzed using independent *t*‐tests, paired *t*‐tests, and Chi‐square. At the end of the study, analysis of covariance (ANCOVA) tests were used to correct the effects of confounding variables and baseline values. Factors, including food intake, energy intake, protein, fat, and carbohydrate diet, physical activity, medication use, duration of HD treatment, number of HD sessions per week, use of products containing probiotic strains in study time, age, sex, weight, height, BMI, occupation, education level, and smoking, were considered as confounders. A significance level below 0.05 was utilized for all analyses.

## 3 RESULTS

### 3.1 Demographic information

**FIGURE 1:**
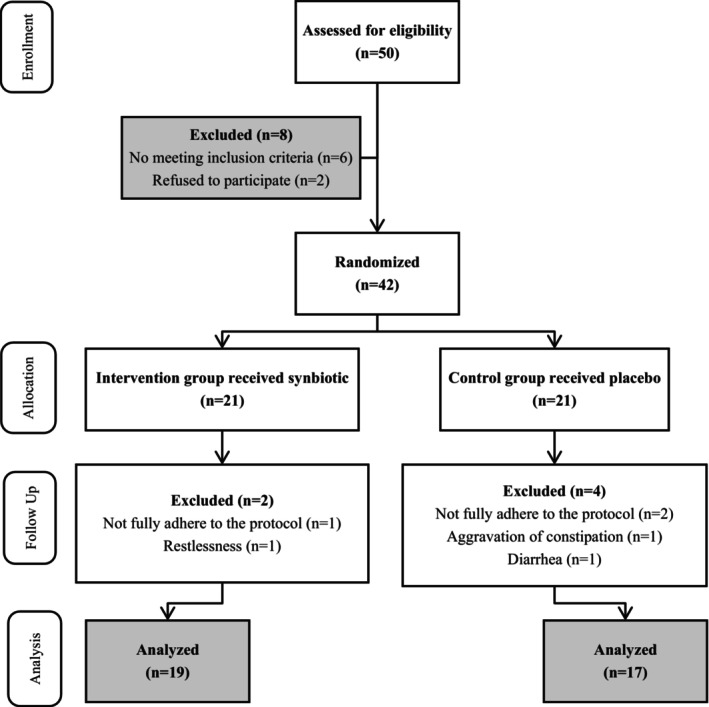
Flow diagram of study.

As depicted in Figure 1, a total of 42 HD patients were ultimately enrolled in the study. The patients were allocated into two distinct groups: intervention (synbiotic) and control, and 21 subjects were randomly assigned to each group. In the SYN group, two patients were excluded from the study due to nonadherence to the protocol and restlessness following supplement intake. Also, in the CON group, four patients were excluded from the study due to not fully adhering to the protocol, constipation, and diarrhea after taking the capsule. Finally, 19 patients in the SYN group and 17 in the CON group were retained until the end of the study and analyzed.

The general characteristics of the HD patients in the two groups are shown in Table 1. Of the total participants, 25 were men (69.4%) and the rest were women. However, in terms of gender distribution, there was no significant difference between the two groups (*p* > .05). The average age of participants was 54.48 ± 16.48 years. The baseline BMI distribution was normal among the groups (22.9 ± 3.9) (See more details in Table 1). As seen in Table 2, there was no difference in the intake of energy, macronutrients (protein, carbohydrate, fat), and micronutrients between the two groups at the beginning and end of the study **(** *p* > .05), and only the intake of saturated fatty acids (SFAs) in the SYN group after the intervention had significantly increased compared with the beginning of the study (*p* = .03). The description and analysis of the AGEs plasma parameters and other blood factors measured before and after the intervention in Table 3 were reported. The analysis of the data showed that the plasma levels of AGEs in both SYN and CON groups were significantly increased after the intervention compared to the baseline values (*p* < .001 and *p* = .001, respectively). After adjusting for the effect of confounders, there was no significant difference in the AGEs levels between the two groups (*p* > .05).

**TABLE 1:** Baseline demographic characteristics of the study participants.

Group	Synbiotic group (*n* = 19)	Control group (*n* = 17)	*p* Value
Sex (*n*)			
Male	14 (73.7)	11 (64.7)	.07
Female	5 (26.3)	6 (35.3)	
Age* (year)	52.84 ± 17.28	56.12 ± 15.69	.557
Height* (cm)	165 ± 1.4	167 ± 7	.704
Weight* (kg)	64.70 ± 17.86	62.15 ± 14.33	.642
Baseline BMI* (kg/m^2^)	23.42 ± 3.99	22.31 ± 3.84	.402
Physical activity* (MET‐min/Week)	439.53 ± 769.73	432.24 ± 757.79	.949
Diabetes	6 (31.6)	6 (35.3)	.063
Cardiovascular disease	9 (47.4)	10 (58.8)	.739
Smoking	8 (42.1)	2 (11.8)	.8
Duration of hemodialysis* (h)	3.89 ± 0.32	3.82 ± 0.40	.551
Duration of hemodialysis^2^ (mo)	56.42 ± 41.90	59.65 ± 31.52	.798

Abbreviation: BMI, Body mass index.

* Data presented as mean ± SD and analyzed using *t*‐test, except indicated with * as number (percent) with chi‐square.

**TABLE 2:** Comparison of baseline and final dietary intakes of participants.

Group	Synbiotic group (*n* = 19)			Control group (*n* = 17)				
Variable	Before	After	*p* ^a^	Before	After	*p* ^a^	p**	p***
Energy (kcal/day)	2082.94 ± 439.78	2115.39 ± 406.31	.235	2139.74 ± 414.51	2149.94 ± 419.93	.692	.693	.803
Protein (g/day)	80.24 ± 22.95	82.40 ± 19.23	.286	82.70 ± 18.26	82.46 ± 14.78	.904	.726	.991
Carbohydrate (g/day)	267.97 ± 85.02	266.39 ± 73.59	.699	262.05 ± 71.72	268.32 ± 74.56	.167	.823	.938
Fat (g/day)	76.67 ± 17.86	80.02 ± 16.49	.175	84.52 ± 19.18	82.97 ± 20.62	.24	.212	.636
SFAs (g/day)	19.65 ± 5.11	21.48 ± 5.53	.03	21.31 ± 5.01	21.54 ± 4.8	.76	.333	.973
MUFAs (g/day)	20.86 ± 5.67	20.68 ± 7.16	.889	22.15 ± 6.67	21.15 ± 5.55	.3	.535	.83
PUFAs (g/day)	29.46 ± 9.57	31.37 ± 7.80	.145	34.34 ± 10.76	33.69 ± 12.87	.548	.159	.513
Cholesterol (mg/day)	278.7 ± 114.7	283.3 ± 70.2	.875	277.3 ± 134.1	285.9 ± 101.2	.774	.973	.901
Sodium (mg/day)	764.25 ± 613.3	814.35 ± 513.7	.459	776.74 ± 404.8	695.31 ± 402.6	.264	.943	.448
Potassium (mg/day)	1789.8 ± 425.6	1883.6 ± 474.8	.0708	1626.7 ± 497.4	1640.7 ± 511	.847	.295	.148
Calcium (mg/day)	468.02 ± 179.5	527.10 ± 205.1	.187	452.71 ± 119.9	503.24 ± 205.1	.256	.768	.729
Phosphorus (mg/day)	769.56 ± 242.2	763.36 ± 243.1	.857	863.33 ± 434.5	858.38 ± 396.5	.852	.425	.386

*Note*: Data presented as mean ± SD. Independent sample *t*‐test, used for between‐group comparisons of baseline ^**^ and endpoint *** values.

Abbreviations: MUFAs, monounsaturated fatty acids; PUFAs, polyunsaturated fatty acids; SFAs, saturated fatty acids.

^a^ Paired *t*‐test used for within‐group comparisons.

**TABLE 3:** Hemodialysis' baseline and endpoint blood parameters in the treatment and control groups.

Group	Synbiotic group (*n* = 19)			Control group (*n* = 17)				
Variable	Before	After	*p* *	Before	After	*p* *	*p* **	*p* ***
AGEs (ng/L)	711.96 ± 248.49	870.51 ± 300.78	<.001	741.36 ± 272.80	847.98 ± 245.36	.001	.737	.272
Fibrinogen (mg/dL)	335.74 ± 56.65	302.42 ± 50.56	.007	312.47 ± 67.78	322.18 ± 61.42	.442	.269	.016
HbA1c (%)	6.44 ± 1.57	6.54 ± 1.77	.517	7.20 ± 2.20	7.08 ± 1.91	.7	.235	.991
Hemoglobin (g/dL)	11.10 ± 1.54	10.98 ± 1.68	.669	11.05 ± 1.46	11.14 ± 1.24	.797	.922	.832
FBS (mg/dL)	88.17 ± 22.16	92.35 ± 21.61	.370	87.6 ± 17.5	98.06 ± 30.8	.036	.936	.217
BUN (mg/dL)	66.70 ± 15.03	52.70 ± 11.21	.003	71.06 ± 13.11	60.46 ± 13.03	.007	.391	.116
Creatinine (mg/dL)	8.55 ± 2.15	7.87 ± 2.59	.147	9.38 ± 1.84	8.88 ± 2.22	.304	.252	.610
Sodium (meq/dL)	136.58 ± 4.41	139.23 ± 3.78	.053	138.93 ± 2.89	139.66 ± 3.15	.32	.09	.842
Potassium (mmol/L)	5.19 ± 1.10	5.00 ± 0.68	.471	5.11 ± 0.69	5.13 ± 0.64	.926	.808	.523
Calcium (mg/dL)	8.45 ± 0.73	8.69 ± 0.63	.23	8.63 ± 0.365	8.65 ± 0.418	.851	.414	.476
Phosphorus (mg/dL)	5.15 ± 1.07	5.16 ± 1.03	.982	5.6 ± 0.95	5.57 ± 1.33	.921	.234	.716
Cholesterol (mg/dL)	122.30 ± 21.66	113.84 ± 24.84	.192	136.66 ± 42.12	140.41 ± 53.99	.621	.289	.249
Triglycerides (mg/dL)	142.5 ± 68.26	136.64 ± 70.28	.781	133.33 ± 57.89	144.58 ± 63.94	.237	.797	.662

*Note*: Data presented as mean ± SD.

Abbreviations: AGEs, Advanced glycation end products; BUN, Blood urea nitrogen; FBS, Fasting blood sugar; HbA1c, Hemoglobin A1c.

* Paired *t*‐test was used for within‐group comparisons.

** Independent sample *t*‐test was used for between‐group comparisons of baseline values.

*** Analysis of covariance (ANCOVA) was used for between‐group comparisons of endpoint values after adjusting for confounders as well as baseline values.

At the end of the study, the mean plasma levels of fibrinogen decreased significantly in the SYN group (*p* = .007), so that the disparity between the two groups was significant after adjusting for the effect of confounders (*p* = .016). HbA1c levels in the SYN and CON groups did not show any significant difference between the two groups at the end of the study (*p* > .05).

The plasma levels of BUN before dialysis were significantly decreased in both the CON and the SYN groups against the basal values, but a greater decrease was observed in the SYN group (*p* = .007 and *p* = .003, respectively). There was no significant difference between the two groups after adjusting for the effect of confounders (*p* > .05). There were no significant differences between the two groups about FBS, creatinine, cholesterol, triglyceride, hemoglobin, sodium, potassium, calcium, and phosphorus (*p* > .05).

## 4 DISCUSSION

The current study was carried out to evaluate the effect of synbiotic supplementation on plasma levels of advanced glycation end products and cardiovascular risk factors in HD patients. The mean plasma fibrinogen levels at the end of the study decreased significantly in the SYN group. According to this study, the levels of AGEs, FBS, HbA1c, BUN, creatinine, sodium, potassium, calcium, phosphorus, cholesterol, and triglycerides were not significantly different between the two groups.

In the present study, no significant difference was seen in the plasma level of AGEs after adjusting for the effect of the confounders. Studies have shown different results in changes in levels of AGEs following prebiotic or probiotic administration. In the Abbasalizad study, the effect of dextrin prebiotic supplements (10 g/day) on AGEs and cardiovascular risk factors in 65 women with type 2 diabetes for 8 weeks was examined. The results showed a significant reduction in AGEs like carboxymethyllysine and malondialdehyde (MDA), as well as an increase in RAGE in the intervention group compared with the placebo. The study was exclusively conducted on overweight patients, with diabetic patients suffering from kidney disorders being excluded from participation (Farhangi et al., 2020).

In a separate study, Mirmiranpour et al. examined the impact of probiotic and synbiotic supplementation on glycemic control in individuals with type 2 diabetes. In the probiotic group, *Lactobacillus acidophilus* (10^8^ CFU) was administered, and in the synbiotic group, combined probiotics and 0.5 g of cinnamon were given to the patients. After 3 months of the study, AGEs in the synbiotic group decreased significantly. However, the study did not report the method for calculating AGEs or the exact plasma or serum levels. Additionally, the statistical method of data analysis was different (Mirmiranpour et al., 2020). Mafi et al.'s study findings demonstrated a noteworthy reduction in serum AGEs levels following probiotic supplementation in patients with diabetic nephropathy. The probiotics group consumed a combination of supplements containing *Lactobacillus* and *Bifidobacteria* (8 × 10^9^ CFU) for 12 weeks. In this study, the statistical method for adjusting for the effect of confounding and the method for measuring serum levels of AGEs (fluorometric method) were different (Mafi et al., 2018). AGEs can initiate a series of reactive oxygen species (ROS) production in various cell types by interacting with RAGE, resulting in elevated oxidative stress (Ohtsu et al., 2017). Probiotics may be considered to reduce oxidative stress by lowering blood sugar and insulin resistance (Aliasgharzadeh et al., 2015), both of which are involved in the formation of AGEs. Therefore, one way that probiotics may reduce plasma levels of AGEs is by decreasing the levels of FBS and HbA1c. Nonetheless, in the current study, a decline in these indicators was not observed at the end of the study in HD patients.

Plasma levels of fibrinogen decreased specifically within the synbiotic group, and a statistically significant disparity between the groups persisted at the study's conclusion. There are few studies on the effects of synbiotic, probiotic, and prebiotic supplements on fibrinogen in different diseases. In our studies, we have found no evidence in this regard in renal patients. In line with the findings of the present study, Naruszewicz et al. observed a notable decrease in fibrinogen and other cardiovascular factors in the intervention group compared to the placebo after 6 weeks of consuming a beverage containing *Lactobacillus plantarum* bacteria (5 × 10^7^ CFU/mL) (Naruszewicz et al., 2002). In another study, Ryan et al. found that the daily administration of two probiotic supplements containing 5.6 × 10^10^ CFU of *Saccharomyces boulardii* in hypercholesterolemic patients did not affect the levels of plasma fibrinogen (Ryan et al., 2015). Also, in the study by Cavallini et al., the consumption of a soy probiotic product with isoflavones did not have any effect on plasma fibrinogen levels in hypercholesterolemic patients. In the mentioned study, patients consumed *Lactobacillus helveticus* and *Enterococcus faecium* (10^10^ CFU) in the form of a soy beverage and 50 mg of isoflavones for 42 days (Cavallini et al., 2016). In contrast to the findings of the present study, Larsen et al. conducted a study to examine the impact of probiotic milk on overweight and obese individuals. The researchers demonstrated that of *Enterococcus faecium* and *Streptococcus thermophilus* elevated plasma fibrinogen levels. It is plausible that the impact on fibrinogen could be attributed to immune stimulation by one of the bacteria in the product (Agerholm‐Larsen et al., 2000). The diversity of strains and the dose of bacteria present in the probiotic may be the reasons for the differences in the studies. According to the evidence, lactobacilli are more likely to affect fibrinogen; however, the mentioned hypothesis needs further examination in studies.

In the present study, the HbA1c and FBS levels did not change in the groups at the end of the study. Findings from various studies have indicated that probiotics have a beneficial impact on lowering FBS and HbA1C levels, although certain studies have not detected any significant effect following the intervention. In their study, Jiang et al. observed that following a 12‐week regimen of a synbiotic supplement in 76 patients with diabetic nephropathy, the levels of FBS and HbA1C decreased in both the SYN and CON groups. However, the decrease in HbA1C levels was statistically significant only in the synbiotic group (Jiang et al., 2021). Furthermore, in the meta‐analysis conducted by Zarezadeh et al. (2022) the probiotic supplement resulted in a substantial reduction in Fasting Plasma Glucose (FPG), HbA1c, and insulin levels in patients. Dixon et al.'s study indicates that the impact of probiotics on lowering glucose and HbA1c levels varies across different conditions. For example, individuals with diabetes may experience different outcomes based on the dosage and duration of probiotic consumption (Dixon et al., 2020). Various studies have shown varying effects of probiotic bacteria on the glycemic index, necessitating the evaluation of different strains and doses of bacteria.

The mean levels of BUN decreased in both groups, but there was no difference between the two groups at the end of the study. Mirzaeian et al. demonstrated that synbiotic supplementation did not yield significant effects on serum levels of urea, creatinine, liver enzymes, high‐sensitivity C‐reactive protein (hsCRP), sodium, potassium, phosphorus, blood pressure, and albumin in HD (Mirzaeian et al., 2020). Furthermore, Mitrovic et al. discovered that synbiotic supplementation did not yield a statistically significant impact on BUN and potassium indicators at the study's conclusion (Mitrović et al., 2023). Firouzi's meta‐analysis of probiotics and kidney function blood parameters revealed that probiotics led to a nonsignificant rise in creatinine and a significant reduction in BUN, particularly when the intervention lasted for fewer than 12 weeks and multistrain probiotics were utilized (Firouzi & Haghighatdoost, 2018). Most studies evaluating the influence of probiotics on Cr and BUN levels have focused on patients with diabetes. Based on the evidence, the effect of probiotics on Cr and BUN levels in kidney patients has a different mechanism. When evaluating the effectiveness of probiotics in chronic renal disease, it is important to consider the stage of CKD. According to Borges et al. studies (Borges et al., 2018; Hyun et al., 2013), there is less possibility that the administration of probiotics can improve biomarkers and uremic toxins in dialysis patients and patients with renal disease without any performance.

This study was the first clinical trial that investigated the effects of synbiotics on AGEs and fibrinogen in HD patients. The main strength of the present study was the period of follow‐up. However, some limitations of this study need to be discussed. First, it was not possible to determine the dietary intake or the effect of food intake on the plasma levels of AGEs. Second, due to budget constraints, microbial culture of feces was not possible for a complete examination.

## 5 CONCLUSION

According to the results of this study, the intake of synbiotic supplementation after 12 weeks led to an improvement in plasma fibrinogen levels and BUN in HD patients, but it did not affect the plasma levels of AGEs, HbA1c, FBS, cholesterol, triglycerides, creatinine, sodium, potassium, calcium, and phosphorus. According to studies, women suffer from gut microbiome disorders to a greater extent. Further research is required to investigate the effect of probiotics on AGEs and other factors affecting cardiovascular diseases in different target groups such as the same gender and at different stages of renal disease.

## AUTHOR CONTRIBUTIONS

**Yasaman Azamian:** Data curation (equal); investigation (equal); project administration (equal); resources (equal); validation (equal); visualization (equal); writing – original draft (equal); writing – review and editing (equal). **Hadi Abdollahzad:** Conceptualization (lead); funding acquisition (lead); investigation (lead); methodology (equal); project administration (equal); resources (equal); supervision (lead); writing – original draft (equal); writing – review and editing (equal). **Shahab Rezaeian:** Data curation (equal); formal analysis (equal); investigation (equal); methodology (equal); software (equal); validation (equal); writing – review and editing (equal). **Mohammad Hossein Rouhani:** Project administration (equal); resources (equal); writing – original draft (equal); writing – review and editing (equal). **Mohammad Hossein Fatehi:** Project administration (equal); writing – original draft (equal); writing – review and editing (equal).

## FUNDING INFORMATION

The present study was supported by the Vice Chancellor for Research and Technology Committee of Kermanshah University of Medical Sciences (grant number 4010134).

## CONFLICT OF INTEREST STATEMENT

The authors affirm that they have no conflicts of interest.

## ETHICS STATEMENTS

This study was approved by the Ethics Committee of Kermanshah University of Medical Sciences (IR.KUMS.REC.1401.033) and registered with the Iranian Clinical Trial Study System (IRCT20131013014994N7).

## INFORMED CONSENT

All study participants provided written informed consent.

## CONSENT FOR PUBLICATION

All authors have granted their consent for the publication of this manuscript.

## Data Availability

The data that support the findings of this study are available on request from the first author (E‐mail: yasaman_azamian@yahoo.com).

## References

[fsn34338-bib-0001] Agerholm‐Larsen, L. , Raben, A. , Haulrik, N. , Hansen, A. S. , Manders, M. , & Astrup, A. (2000). Effect of 8 week intake of probiotic milk products on risk factors for cardiovascular diseases. European Journal of Clinical Nutrition, 54(4), 288–297. 10.1038/sj.ejcn.1600937 10745279

[fsn34338-bib-0002] Alamir, I. , Niquet‐Leridon, C. , Jacolot, P. , Rodriguez, C. , Orosco, M. , Anton, P. M. , & Tessier, F. J. (2013). Digestibility of extruded proteins and metabolic transit of N ε‐carboxymethyllysine in rats. Amino Acids, 44(6), 1441–1449. 10.1007/s00726-012-1427-3 23160731

[fsn34338-bib-0003] Aliasgharzadeh, A. , Khalili, M. , Mirtaheri, E. , Gargari, B. P. , Tavakoli, F. , Farhangi, M. A. , Babaei, H. , & Dehghan, P. (2015). A combination of prebiotic inulin and oligofructose improve some of cardiovascular disease risk factors in women with type 2 diabetes: A randomized controlled clinical trial. Advanced Pharmaceutical Bulletin, 5(4), 507. 10.15171/apb.2015.069 26819923 PMC4729356

[fsn34338-bib-0004] Bakhtiary, M. , Morvaridzadeh, M. , Agah, S. , Rahimlou, M. , Christopher, E. , Zadro, J. R. , & Heshmati, J. (2021). Effect of probiotic, prebiotic, and Synbiotic supplementation on cardiometabolic and oxidative stress parameters in patients with chronic kidney disease: A systematic review and meta‐analysis. Clinical Therapeutics, 43, e71–e96. 10.1016/j.clinthera.2020.12.021 33526314

[fsn34338-bib-0005] Bierhaus, A. , Hofmann, M. A. , Ziegler, R. , & Nawroth, P. P. (1998). AGEs and their interaction with AGE‐receptors in vascular disease and diabetes mellitus. I. The AGE concept. Cardiovascular Research, 37(3), 586–600. 10.1016/s0008-6363(97)00233-2 9659442

[fsn34338-bib-0006] Borges, N. A. , Carmo, F. L. , Stockler‐Pinto, M. B. , De Brito, J. S. , Dolenga, C. J. , Ferreira, D. C. , Nakao, L. S. , Rosado, A. , Fouque, D. , & Mafra, D. (2018). Probiotic supplementation in chronic kidney disease: A double‐blind, randomized, placebo‐controlled trial. Journal of Renal Nutrition, 28(1), 28–36. 10.1053/j.jrn.2017.06.010 28888762

[fsn34338-bib-0007] Cavallini, D. C. , Manzoni, M. S. , Bedani, R. , Roselino, M. N. , Celiberto, L. S. , Vendramini, R. C. , de Valdez, G. F. , Abdalla, D. S. P. , Pinto, R. A. , Rosetto, D. , Valentini, S. R. , & Rossi, E. A. (2016). Probiotic soy product supplemented with isoflavones improves the lipid profile of moderately hypercholesterolemic men: A randomized controlled trial. Nutrients, 8(1), 52. 10.3390/nu8010052 26797632 PMC4728664

[fsn34338-bib-0008] Cozzolino, M. , Galassi, A. , Pivari, F. , Ciceri, P. , & Conte, F. (2017). The cardiovascular burden in end‐stage renal disease. Expanded Hemodialysis, 191, 44–57. 10.1159/000479250 28910790

[fsn34338-bib-0009] Dixon, A. , Robertson, K. , Yung, A. , Que, M. , Randall, H. , Wellalagodage, D. , Cox, T. , Robertson, D. , Chi, C. , & Sun, J. (2020). Efficacy of probiotics in patients of cardiovascular disease risk: A systematic review and meta‐analysis. Current Hypertension Reports, 22(9), 74. 10.1007/s11906-020-01080-y 32860083

[fsn34338-bib-0010] Farhangi, M. A. , Dehghan, P. , & Namazi, N. (2020). Prebiotic supplementation modulates advanced glycation end‐products (AGEs), soluble receptor for AGEs (sRAGE), and cardiometabolic risk factors through improving metabolic endotoxemia: A randomized‐controlled clinical trial. European Journal of Nutrition, 59, 3009–3021. 10.1007/s00394-019-02140-z 31728681

[fsn34338-bib-0011] Firouzi, S. , & Haghighatdoost, F. (2018). The effects of prebiotic, probiotic, and synbiotic supplementation on blood parameters of renal function: A systematic review and meta‐analysis of clinical trials. Nutrition, 51, 104–113. 10.1016/j.nut.2018.01.007 29626749

[fsn34338-bib-0012] Gansevoort, R. T. , Correa‐Rotter, R. , Hemmelgarn, B. R. , Jafar, T. H. , Heerspink, H. J. L. , Mann, J. F. , Matsushita, K. , & Wen, C. P. (2013). Chronic kidney disease and cardiovascular risk: Epidemiology, mechanisms, and prevention. The Lancet, 382(9889), 339–352. 10.1016/S0140-6736(13)60595-4 23727170

[fsn34338-bib-0013] Gugliucci, A. , & Bendayan, M. (1996). Renal fate of circulating advanced glycated end products (AGE): Evidence for reabsorption and catabolism of AGE‐peptides by renal proximal tubular cells. Diabetologia, 39(2), 149–160. 10.1007/BF00403957 8635666

[fsn34338-bib-0014] Hallal, P. C. , & Victora, C. G. (2004). Reliability and validity of the international physical activity questionnaire (IPAQ). Medicine and Science in Sports and Exercise, 36(3), 556. 10.1249/01.mss.0000117161.66394.07 15076800

[fsn34338-bib-0015] Henning, C. , & Glomb, M. A. (2016). Pathways of the Maillard reaction under physiological conditions. Glycoconjugate Journal, 33(4), 499–512. 10.1007/s10719-016-9694-y 27291759

[fsn34338-bib-0016] Herzog, C. A. , Asinger, R. W. , Berger, A. K. , Charytan, D. M. , Díez, J. , Hart, R. G. , Eckardt, K.‐U. , Kasiske, B. L. , McCullough, P. A. , Passman, R. S. , DeLoach, S. S. , Pun, P. H. , & Passman, R. S. (2011). Cardiovascular disease in chronic kidney disease. A clinical update from kidney disease: Improving global outcomes (KDIGO). Kidney International, 80(6), 572–586. 10.1038/ki.2011.223 21750584

[fsn34338-bib-0017] Hyun, H. S. , Paik, K. H. , & Cho, H. Y. (2013). p‐Cresyl sulfate and indoxyl sulfate in pediatric patients on chronic dialysis. Korean Journal of Pediatrics, 56(4), 159–164. 10.3345/kjp.2013.56.4.159 23646054 PMC3641312

[fsn34338-bib-0018] Jazani, N. H. , Savoj, J. , Lustgarten, M. , Lau, W. L. , & Vaziri, N. D. (2019). Impact of gut dysbiosis on neurohormonal pathways in chronic kidney disease. Diseases, 7(1), 21. 10.3390/diseases7010021 30781823 PMC6473882

[fsn34338-bib-0019] Jiang, H. , Zhang, Y. , Xu, D. , & Wang, Q. (2021). Probiotics ameliorates glycemic control of patients with diabetic nephropathy: A randomized clinical study. Journal of Clinical Laboratory Analysis, 35(4), e23650. 10.1002/jcla.23650 33666270 PMC8059722

[fsn34338-bib-0020] Joossens, M. , Faust, K. , Gryp, T. , Nguyen, A. T. L. , Wang, J. , Eloot, S. , Schepers, E. , Dhondt, A. , Pletinck, A. , Vieira‐Silva, S. , Falony, G. , Vaneechoutte, M. , Vanholder, R. , Van Biesen, W. , Huys, G. R. B. , Raes, J. , & Vieira‐Silva, S. (2019). Gut microbiota dynamics and uraemic toxins: One size does not fit all. Gut, 68(12), 2257–2260. 10.1136/gutjnl-2018-317561 PMC6872439 30464044

[fsn34338-bib-0021] Koppe, L. , Mafra, D. , & Fouque, D. (2015). Probiotics and chronic kidney disease. Kidney International, 88(5), 958–966. 10.1038/ki.2015.255 26376131

[fsn34338-bib-0022] Koschinsky, T. , He, C.‐J. , Mitsuhashi, T. , Bucala, R. , Liu, C. , Buenting, C. , Heitmann, K. , & Vlassara, H. (1997). Orally absorbed reactive glycation products (glycotoxins): An environmental risk factor in diabetic nephropathy. Proceedings of the National Academy of Sciences of the United States of America, 94(12), 6474–6479. 10.1073/pnas.94.12.6474 9177242 PMC21074

[fsn34338-bib-0023] Levey, A. S. , Becker, C. , & Inker, L. A. (2015). Glomerular filtration rate and albuminuria for detection and staging of acute and chronic kidney disease in adults: A systematic review. JAMA, 313(8), 837–846. 25710660 10.1001/jama.2015.0602 PMC4410363

[fsn34338-bib-0024] Mafi, A. , Namazi, G. , Soleimani, A. , Bahmani, F. , Aghadavod, E. , & Asemi, Z. (2018). Metabolic and genetic response to probiotics supplementation in patients with diabetic nephropathy: A randomized, double‐blind, placebo‐controlled trial. Food & Function, 9(9), 4763–4770. 10.1039/c8fo00888d 30113051

[fsn34338-bib-0025] Mirmiranpour, H. , Huseini, H. F. , Derakhshanian, H. , Khodaii, Z. , & Tavakoli‐Far, B. (2020). Effects of probiotic, cinnamon, and synbiotic supplementation on glycemic control and antioxidant status in people with type 2 diabetes; a randomized, double‐blind, placebo‐controlled study. Journal of Diabetes & Metabolic Disorders, 19, 53–60. 10.1007/s40200-019-00474-3 32550156 PMC7270449

[fsn34338-bib-0026] Mirzaeian, S. , Saraf‐Bank, S. , Entezari, M. H. , Hekmatdoost, A. , Feizi, A. , & Atapour, A. (2020). Effects of synbiotic supplementation on microbiota‐derived protein‐bound uremic toxins, systemic inflammation, and biochemical parameters in patients on hemodialysis: A double‐blind, placebo‐controlled, randomized clinical trial. Nutrition, 73, 110713. 10.1016/j.nut.2019.110713 32120316

[fsn34338-bib-0027] Mitrović, M. , Stanković‐Popović, V. , Tolinački, M. , Golić, N. , Soković Bajić, S. , Veljović, K. , Nastasijević, B. , Soldatović, I. , Svorcan, P. , & Dimković, N. (2023). The impact of Synbiotic treatment on the levels of gut‐derived uremic toxins, inflammation, and gut microbiome of chronic kidney disease patients‐a randomized trial. Journal of Renal Nutrition, 33(2), 278–288. 10.1053/j.jrn.2022.07.008 35995418

[fsn34338-bib-0028] Miyata, T. , De Strihou, C. V. Y. , Kurokawa, K. , & Baynes, J. W. (1999). Alterations in nonenzymatic biochemistry in uremia: Origin and significance of “carbonyl stress” in long‐term uremic complications. Kidney International, 55(2), 389–399. 10.1046/j.1523-1755.1999.00302.x 9987064

[fsn34338-bib-0029] Miyata, T. , Ueda, Y. , Horie, K. , Nangaku, M. , Tanaka, S. , De Strihou, C. V. Y. , & Kurokawa, K. (1998). Renal catabolism of advanced glycation end products: The fate of pentosidine. Kidney International, 53(2), 416–422. 10.1046/j.1523-1755.1998.00756.x 9461101

[fsn34338-bib-0030] Naruszewicz, M. , Johansson, M. L. , Zapolska‐Downar, D. , & Bukowska, H. (2002). Effect of lactobacillus plantarum 299v on cardiovascular disease risk factors in smokers. The American Journal of Clinical Nutrition, 76(6), 1249–1255. 10.1093/ajcn/76.6.1249 12450890

[fsn34338-bib-0031] Ohtsu, A. , Shibutani, Y. , Seno, K. , Iwata, H. , Kuwayama, T. , & Shirasuna, K. (2017). Advanced glycation end products and lipopolysaccharides stimulate interleukin‐6 secretion via the RAGE/TLR4‐NF‐κB‐ROS pathways and resveratrol attenuates these inflammatory responses in mouse macrophages. Experimental and Therapeutic Medicine, 14(5), 4363–4370. 10.3892/etm.2017.5045 29067115 PMC5647727

[fsn34338-bib-0032] Raj, D. S. , Choudhury, D. , Welbourne, T. C. , & Levi, M. (2000). Advanced glycation end products: A Nephrologist's perspective. American Journal of Kidney Diseases, 35(3), 365–380. 10.1016/s0272-6386(00)70189-2 10692262

[fsn34338-bib-0033] Ramezani, A. , Massy, Z. A. , Meijers, B. , Evenepoel, P. , Vanholder, R. , & Raj, D. S. (2016). Role of the gut microbiome in uremia: A potential therapeutic target. American Journal of Kidney Diseases, 67(3), 483–498. 10.1053/j.ajkd.2015.09.027 26590448 PMC5408507

[fsn34338-bib-0034] Ramezani, A. , & Raj, D. S. (2014). The gut microbiome, kidney disease, and targeted interventions. Journal of the American Society of Nephrology, 25(4), 657–670. 10.1681/ASN.2013080905 24231662 PMC3968507

[fsn34338-bib-0035] Ryan, J. J. , Hanes, D. A. , Schafer, M. B. , Mikolai, J. , & Zwickey, H. (2015). Effect of the probiotic saccharomyces boulardii on cholesterol and lipoprotein particles in hypercholesterolemic adults: A single‐arm, open‐label pilot study. The Journal of Alternative and Complementary Medicine, 21(5), 288–293. 10.1089/acm.2014.0063 25893960 PMC4432884

[fsn34338-bib-0036] Schepers, E. , Glorieux, G. , & Vanholder, R. (2010). The gut: The forgotten organ in uremia? Blood Purification, 29(2), 130–136. 10.1159/000245639 20093818

[fsn34338-bib-0037] Schmidt, A. M. , & Stern, D. (2000). Atherosclerosis and diabetes: The RAGE connection. Current Atherosclerosis Reports, 2(5), 430–436. 10.1007/s11883-000-0082-4 11122775

[fsn34338-bib-0038] Sesso, R. C. , Lopes, A. A. , Thomé, F. S. , Lugon, J. R. , Watanabe, Y. , & Santos, D. R. D. (2014). Report of the Brazilian chronic dialysis census 2012. Brazilian Journal of Nephrology, 36, 48–53. 10.5935/0101-2800.20140009 24676614

[fsn34338-bib-0039] Strid, H. , Simrén, M. , Stotzer, P.‐O. , Ringström, G. , Abrahamsson, H. , & Björnsson, E. S. (2003). Patients with chronic renal failure have abnormal small intestinal motility and a high prevalence of small intestinal bacterial overgrowth. Digestion, 67(3), 129–137. 10.1159/000071292 12853724

[fsn34338-bib-0040] Vaziri, N. D. , Wong, J. , Pahl, M. , Piceno, Y. M. , Yuan, J. , DeSantis, T. Z. , Ni, Z. , Nguyen, T.‐H. , & Andersen, G. L. (2013). Chronic kidney disease alters intestinal microbial flora. Kidney International, 83(2), 308–315. 10.1038/ki.2012.345 22992469

[fsn34338-bib-0041] Ward, M. S. , Fotheringham, A. K. , Cooper, M. E. , & Forbes, J. M. (2013). Targeting advanced glycation endproducts and mitochondrial dysfunction in cardiovascular disease. Current Opinion in Pharmacology, 13(4), 654–661. 10.1016/j.coph.2013.06.009 23871446

[fsn34338-bib-0042] Wu, M.‐J. , Chang, C.‐S. , Cheng, C.‐H. , Chen, C.‐H. , Lee, W.‐C. , Hsu, Y.‐H. , Shu, K.‐H. , & Tang, M.‐J. (2004). Colonic transit time in long‐term dialysis patients. American Journal of Kidney Diseases, 44(2), 322–327. 10.1053/j.ajkd.2004.04.048 15264191

[fsn34338-bib-0043] Yacoub, R. , Nugent, M. , Cai, W. , Nadkarni, G. N. , Chaves, L. D. , Abyad, S. , Honan, A. M. , Thomas, S. A. , Zheng, W. , Valiyaparambil, S. A. , Bryniarski, M. A. , Sun, Y. , Buck, M. , Genco, R. J. , Quigg, R. J. , He, J. C. , & Valiyaparambil, S. A. (2017). Advanced glycation end products dietary restriction effects on bacterial gut microbiota in peritoneal dialysis patients; a randomized open label controlled trial. PLoS One, 12(9), e0184789. 10.1371/journal.pone.0184789 28931089 PMC5607175

[fsn34338-bib-0044] Yuan, X. , Zhao, J. , Qu, W. , Zhang, Y. , Jia, B. , Fan, Z. , He, Q. , & Li, J. (2018). Accumulation and effects of dietary advanced glycation end products on the gastrointestinal tract in rats. International Journal of Food Science & Technology, 53(10), 2273–2281. 10.1111/ijfs.13817

[fsn34338-bib-0045] Zarezadeh, M. , Musazadeh, V. , Faghfouri, A. H. , Sarmadi, B. , Jamilian, P. , Jamilian, P. , Tutunchi, H. , & Dehghan, P. (2022). Probiotic therapy, a novel and efficient adjuvant approach to improve glycemic status: An umbrella meta‐analysis. Pharmacological Research, 183, 106397. 10.1016/j.phrs.2022.106397 35981707

[fsn34338-bib-0046] Zheng, H. J. , Guo, J. , Wang, Q. , Wang, L. , Wang, Y. , Zhang, F. , Huang, W.‐J. , Zhang, W. , Liu, W. J. , & Wang, Y. (2021). Probiotics, prebiotics, and synbiotics for the improvement of metabolic profiles in patients with chronic kidney disease: A systematic review and meta‐analysis of randomized controlled trials. Critical Reviews in Food Science and Nutrition, 61(4), 577–598. 10.1080/10408398.2020.1740645 32329633

[fsn34338-bib-0047] Zimmermann, J. , Herrlinger, S. , Pruy, A. , Metzger, T. , & Wanner, C. (1999). Inflammation enhances cardiovascular risk and mortality in hemodialysis patients. Kidney International, 55(2), 648–658. 10.1046/j.1523-1755.1999.00273.x 9987089

